# Tumor microenvironment B cells increase bladder cancer metastasis *via* modulation of the IL-8/androgen receptor (AR)/MMPs signals

**DOI:** 10.18632/oncotarget.4569

**Published:** 2015-07-17

**Authors:** Zhenyu Ou, Yongjie Wang, Longfei Liu, Lei Li, Shuyuan Yeh, Lin Qi, Chawnshang Chang

**Affiliations:** ^1^ Departments of Urology and Plastic Surgery, Xiangya Hospital, Central South University, Changsha, China; ^2^ George Whipple Lab for Cancer Research, Departments of Pathology and Urology, University of Rochester Medical Center, Rochester NY, USA; ^3^ Sex hormone Research Center, China Medical University/Hospital, Taichung 404, Taiwan

**Keywords:** bladder cancer, androgen receptor, B cell, tumor microenvironment, tumor metastasis

## Abstract

While B cells in the tumor microenvironment may play important roles in cancer progression, their impacts on the bladder cancer (BCa) metastasis remain unclear. Here we found from human clinical BCa samples that BCa tissues could recruit more B cells than the surrounding normal bladder tissues and the *in vitro* co-culture assay also demonstrated that B cells could be recruited more easily towards BCa cells compared to normal bladder cells. Chamber invasion and 3D invasion assays showed the recruited B cells could then significantly increase the BCa cell invasion. Mechanism dissection found that recruited B cells could increase IL-8/androgen receptor (AR) signals in BCa cells that could then promote the expression of metastasis genes including MMP1 and MMP13. Blocking the IL-8/AR/MMPs signals either by anti-IL-8 neutralizing antibody, AR-siRNA, or MMPs inhibitors all partially reversed the infiltrating B cells capacity to increase the BCa cell invasion. The *in vivo* data from orthotopically xenografted BCa mouse model also confirmed that infiltrating B cells could increase BCa cell invasion *via* increasing AR signals. Together, these results demonstrate the key roles of B cells within the bladder tumor microenvironment that increase the BCa metastasis and may help us to develop the potential therapies *via* targeting these newly identified IL-8/AR/MMPs signals to better battle the BCa progression.

## INTRODUCTION

Urothelial carcinoma of the bladder is the most common malignancy in the urinary tract. In the United States, bladder cancer (BCa) is the twelfth leading cancer in women and the fourth in men, [1] and epidemiology studies demonstrated that the incidence of BCa is nearly four times higher in men than in women. [2] However, the detailed mechanisms remain unclear. Androgen/androgen receptors (AR) signals have been implicated in playing an important role in BCa progression. [3, 4]

Growing evidences indicate that the tumor microenvironment (TME) may play important roles for BCa progression. [5, 6] However, the role of B lymphocytes (B cells) during tumor progression is still controversial. Antigen-presenting B cells can enhance tumor-specific cytotoxic T cell activation, [7] yet B cells can also potentiate chronic inflammation that enhances tumor development. [8, 9] Importantly, conclusive data to elucidate the role of B cells in BCa progression is still lacking.

Here we found that B cells could be recruited more easily into the BCa tissues/cells compared to the surrounding normal bladder tissues/cells and consequences of such increased infiltrating B cells to BCa could then promote the BCa cell invasion *via* modulation of interleukin 8 (IL-8)/AR/Matrix Metalloproteinases (MMPs) signals.

## RESULTS

### B cells were recruited more easily to BCa tissues compared to the surrounding normal bladder tissues in human clinical samples

**Figure 1:**
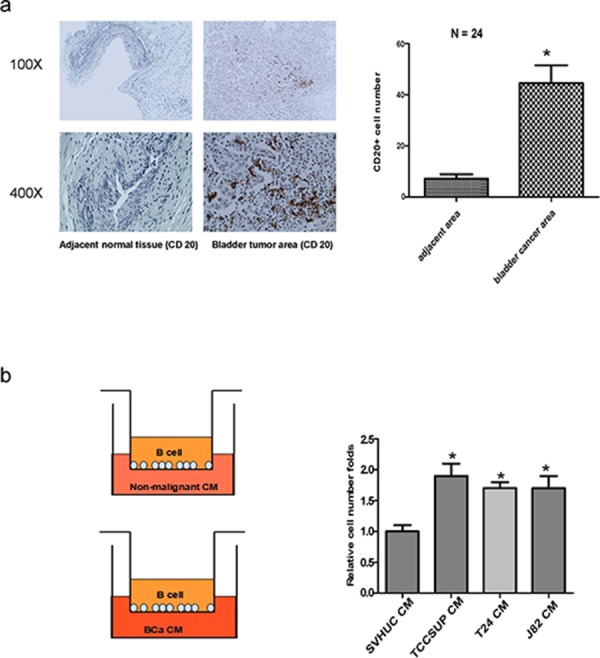
Bladder cancer tissues/cells can better recruit B cells than non-malignant tissues/urothelial cells **a.** More B cells infiltration was noted in BCa tissues compared to adjacent normal bladder area tissues. IHC staining of human bladder tissues was conducted using anti-CD20 antibody (*N* = 24). **b.** Cartoon shows the transwell B cells recruitment assay. Conditioned media (CM) of BCa cells or SVHUC cells was plated into the lower chambers of the transwells. 1 × 10^5^ B cells were plated onto the upper chambers with 5 μm pore polycarbonate membranes. The B cells migrated into the lower chambers were collected after 6 hrs and counted. Data are presented as mean ± SD. *P < 0.05 by student's *t*-test.

Early studies indicated that B cells within the TME were detected in various tumors including BCa. [10] We first applied IHC staining with B cells marker CD20 to compare the B cells infiltration in BCa and their surrounding normal bladder tissues in clinical specimens. The results revealed that more B cells were detected in BCa tissues than adjacent normal bladder tissues (Fig. 1a).

### *In vitro* co-culture system proved B cells were recruited more easily towards BCa cells than normal bladder cells

To confirm the above human clinical data, we applied the *in vitro* co-culture Boyden chamber migration system to compare the capacity of recruiting B cells towards BCa cells vs normal bladder cells. We put the conditioned media (CM) of BCa cells or SVHUC cells in the lower chambers and then placed Ramos B cells onto the upper chambers (Fig. 1b, left panel). After 6 hrs incubation, we counted the number of Ramos B cells that migrated through the membranes into the bottom chambers, and found BCa cells have a much better capacity to recruit the B cells as compared to the non-malignant urothelial SVHUC cells (Fig. 1b, right panel).

Together, results from human clinical BCa samples and *in vitro* cell co-culture system suggest that B cells in TME can be more easily recruited towards the BCa cells than their surrounding normal bladder cells.

### Infiltrating B cells increased BCa cells migration and invasion

**Figure 2:**
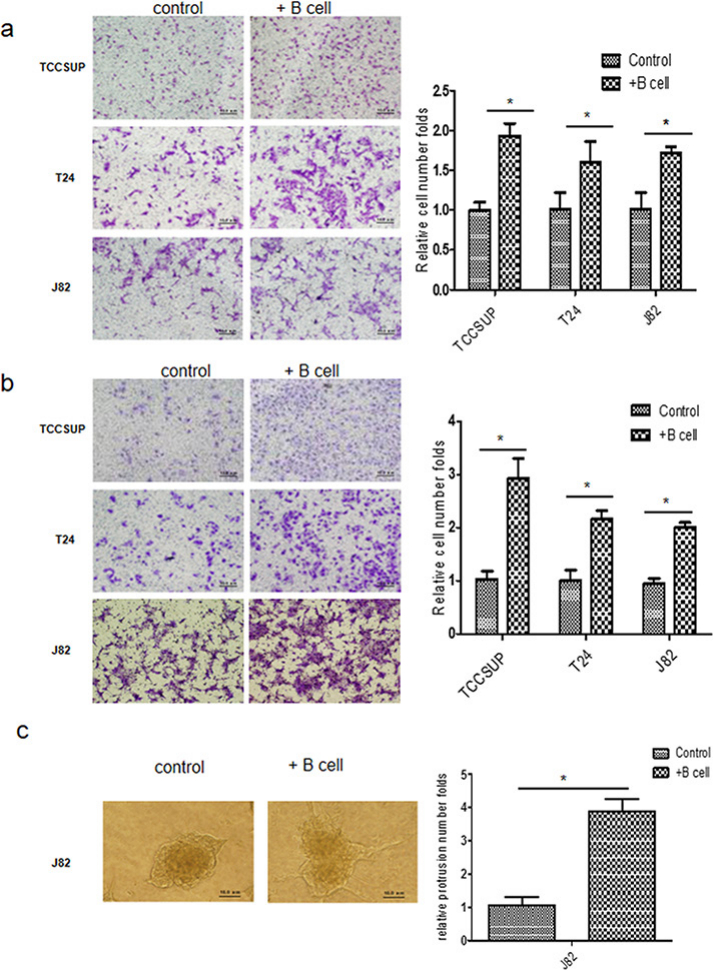
B cells can promote BCa cells migration and invasion **a.** We co-cultured TCCUSP, T24 and J82 cells with B cells for 3 days. The 1 × 10^5^ co-cultured BCa cells were seeded into the upper chambers (with 8 μm size pore) to perform migration assays, 1 × 10^5^ BCa cells without co-culture with B cells were used as controls. After 24 hrs, 0.1% crystal violet blue staining results show BCa cells co-cultured with B cells had a higher invasive capacity as compared to control cells. **b.** BCa cells were subjected to invasion assays using 8 μm size pore chambers coated with matrigel. Image shows BCa cells co-cultured with B cells have a higher ability for migration than BCa cells alone (*P < 0.05). **c.** 3D invasion assay showed that more protrusions structures formed in co-cultured J82 cells than in J82 cells alone. The right panels in A, B, C are the quantification data of left panels. (*P < 0.05).

We then examined the potential impacts of recruitment of more B cells on the BCa progression. We first employed a Chamber co-culture system to assay the BCa cells migration with vs without co-cultured B cells. BCa cell lines (TCCSUP, T24 or J82) were co-cultured with Ramos B cells for 72 hrs before the migration assay, and results revealed that the BCa cell migration was increased significantly after co-culturing with Ramos B cells (Fig. 2a).

The Chamber invasion assay also revealed that co-culturing the BCa cells with Ramos B cells significantly increased the invasion ability of BCa cells (Fig. 2b). We also obtained the similar results when Ramos cells were replaced by U266 cells (supplementary Fig. S1). Importantly, we also obtained similar results (Fig. 2c) when we replaced Chamber invasion assay with the different 3D invasion assay. [11]

Together, results from Figs. 2a–2c and S1 suggest that the infiltrating B cells to the BCa cells may result in increasing the BCa cell invasion.

### Recruited B cells increased BCa cell invasion *via* alteration of AR/MMPs signals

**Figure 3:**
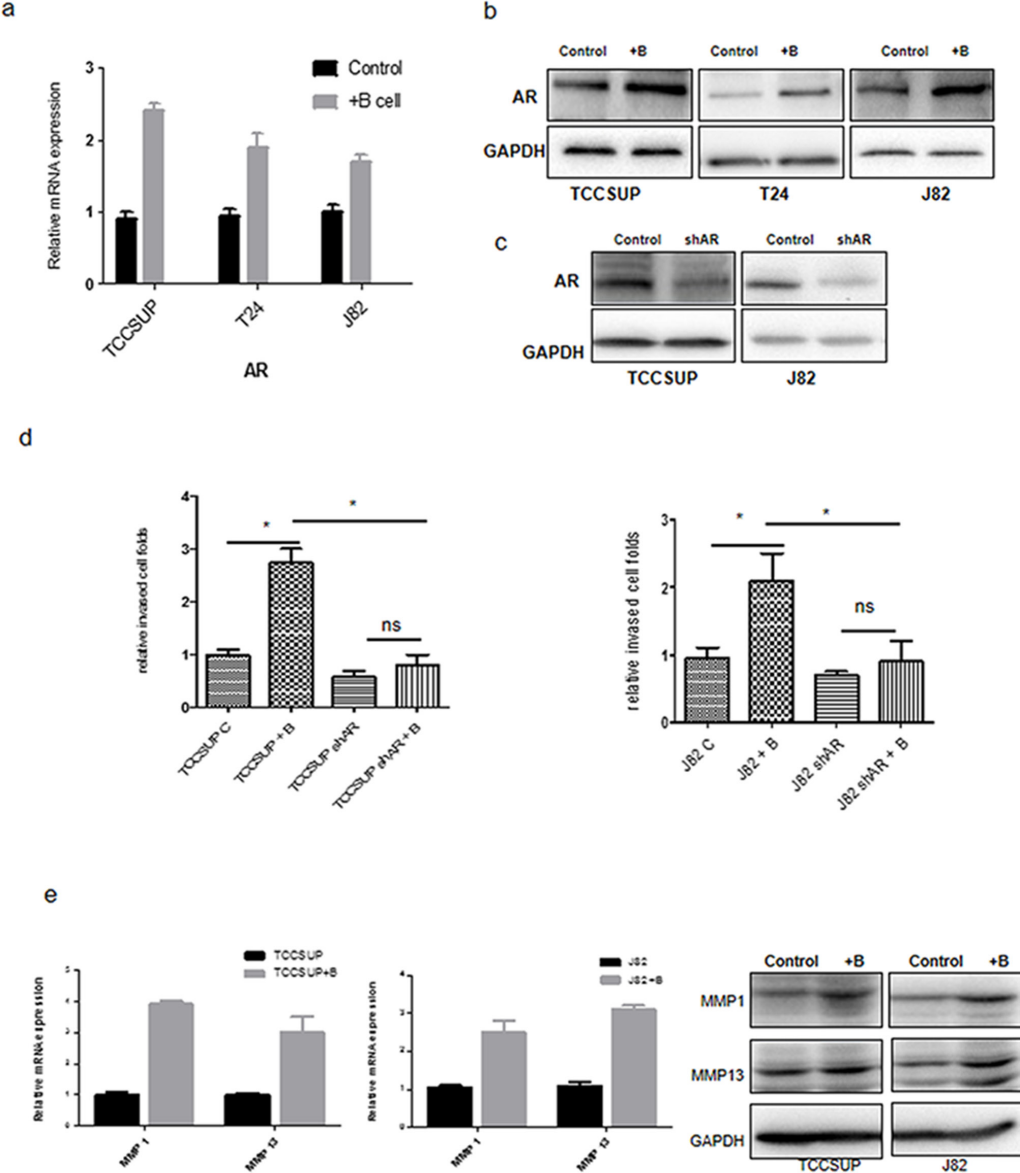

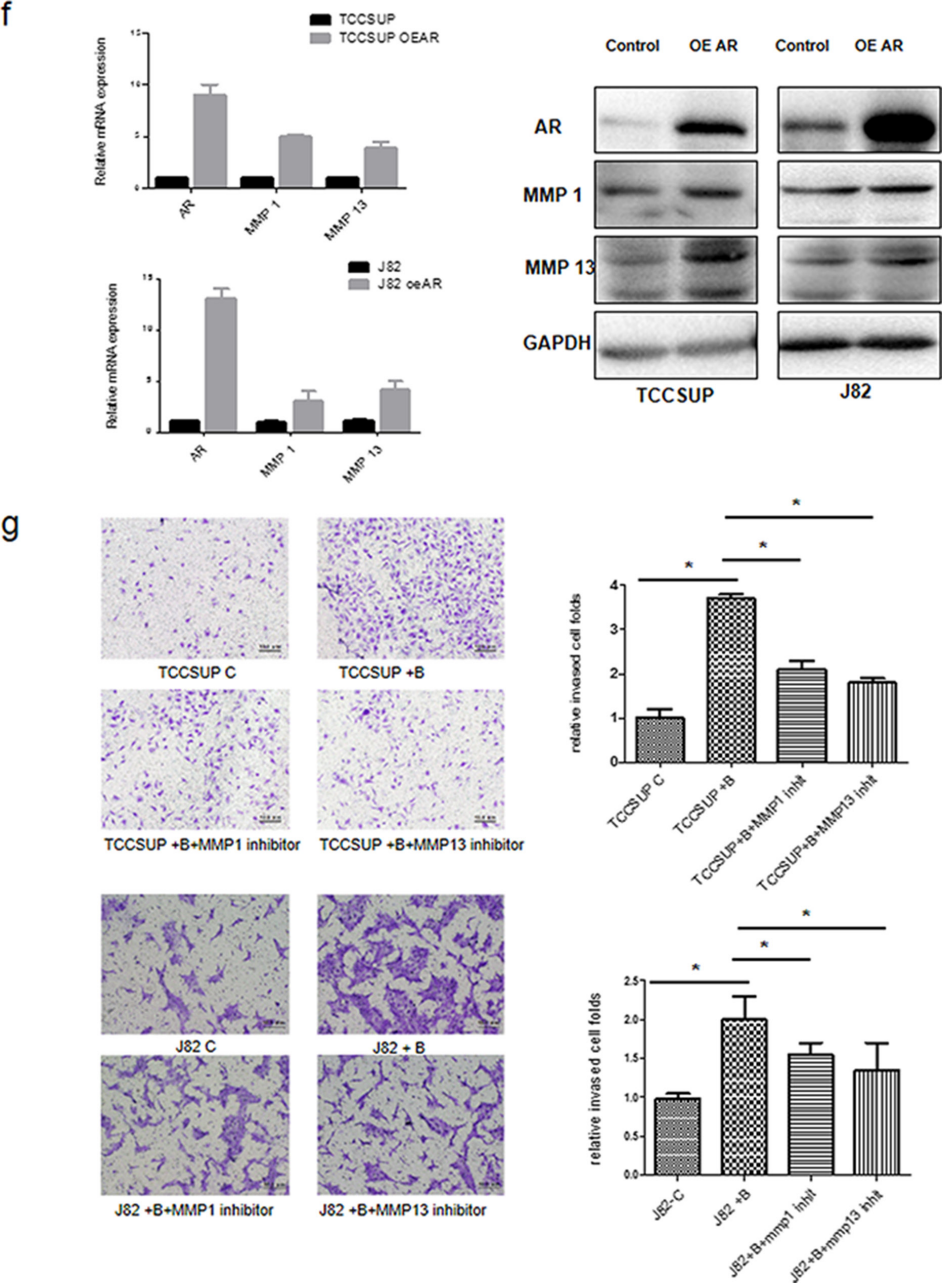
B cells could promote BCa cell invasion via up-regulation of AR/MMP1/MMP13 signaling **a–b.** qRT-PCR (A) and Western blot (B) results showed AR expressions were increased in TCCSUP, T24 and J82 cells after co-culture with B cells. **c.** Validation of AR siRNA knocking down efficiency in TCCSUP and J82 using western blot. **d.** Knocking down AR in BCa cells can reverse the effects of B cells on BCa cell invasion ability. **e.** qRT-PCR and Western blot results show MMP1 and MMP13 expression were increased in TCCSUP and J82 cells after co-culture with B cells. **f.** qRT-PCR (left panels)and Western blot (right panels) results show MMP1 and MMP13 expressions were increased in TCCSUP and J82 cells after overexpressing AR (oeAR). **g.** Invasion assays show B cells promotion of TCCSUP and J82 cells invasion can be partly reversed by MMP1 or MMP13 inhibitor. *P < 0.05 by student's *t*-test. NS: not significant.

To dissect the potential mechanisms by which B cells increased the BCa cell invasion, we first focused on AR signals, which play an important role in BCa progression. [3, 12] Interestingly, we found increased AR expression at both mRNA levels (Fig. 3a) and protein levels (Fig. 3b) in all 3 BCa cell lines (TCCSUP, T24 and J82) co-cultured with B cells.

To further confirm that increased AR plays key roles to mediate the infiltrating B cells-increased BCa cells invasion, we then knocked-down AR expression in BCa cells (Fig. 3c). The results revealed that knocking-down AR in BCa cells reversed the infiltrating B cells capacity to increase BCa cells invasion (Fig. 3d), suggesting infiltrating B cells may function through modulation of AR to increase BCa cell invasion.

To further dissect the molecular mechanisms by which up-regulation of AR in BCa cells increased BCa cell invasion, we examined the expression of several AR downstream metastasis-related genes *via* quantitative PCR assay (supplementary Fig. S2), and found that the expression of MMP1 and MMP13 were significantly increased in TCCSUP and J82 cells after co-culturing with B cells (Fig. 3e). Similar results were obtained when we replaced Ramos cells with U266 cells (supplementary Fig. S3). Adding AR-cDNA in these BCa cells also increased the expression of MMP1 and MMP13 in both mRNA and protein levels (Fig. 3f). We then applied the interruption approach with inhibitors of MMP1 and MMP 13 to see their impacts on AR-increased BCa cell invasion. As expected, we found blocking MMP1 or MMP13 in TCCSUP and J82 cells partially reversed the infiltrating B cells-increased BCa cell invasion (Fig. 3g).

Together, results from Figs. 3, S2 and S3 suggest that infiltrating B cells may function through modulation of AR/MMP1/MMP13 signals in BCa cells to increase BCa cell invasion.

### Mechanism dissection how AR can modulate MMP1 and MMP13 expression

**Figure 4:**
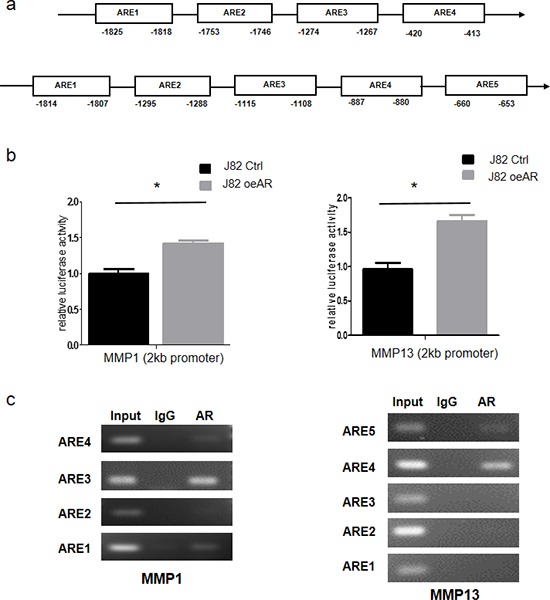
AR transcriptionally up-regulates MMP1 and MMP13 by binding to their promoters **a.** Predicted localization of AREs in MMP1 (top lane) and MMP13 promoter region (bottom lane). **b.** J82-oeAR/vector cells were transfected with MMP1/MMP13-pGL3 luciferase reporter plasmid and pCMVtk vector was used as an internal control for transfection efficiency. After 36 hrs transfection, luciferase activities in triplicate samples were measured. (*P < 0.05) **c.** ChIP analysis of AR binding to the MMP1/MMP13 promoters.

To further dissect the molecular mechanism how AR increases MMP1, MMP13 expression, we first applied PROMO website to identify putative androgen response elements (AREs) in their promoter region. We found 4 or 5 AREs located in the MMP1 or MMP13 2kb promoter regions, respectively (Fig. 4a). We then cloned 2 kb MMP1 and MMP13 promoters into pGL3 vector and applied luciferase reporter assay to examine the AR transaction. The results revealed AR could increase the MMP1 and MMP13 promoter activity in J82 cells (Fig. 4b). We then applied the chromatin immunoprecipitation (ChIP) assay to verify their binding capacity to AR. J82 cells were transfected with the AR-cDNA and then the AR/DNA complex was immunoprecipitated with AR antibodies using IgG antibody as negative control. The results indicated that AR could bind to some of the AREs located within 2kb promoter region of MMP1 and MMP13 (Fig. 4c).

Together, results from Fig. 4a–4c using luciferase reporter assay and ChiP assay demonstrate AR can increase the expression of MMP1 and MMP13 at the transcriptional level.

### Mechanism dissection how infiltrating B cells increased AR expression in BCa cells

**Figure 5:**
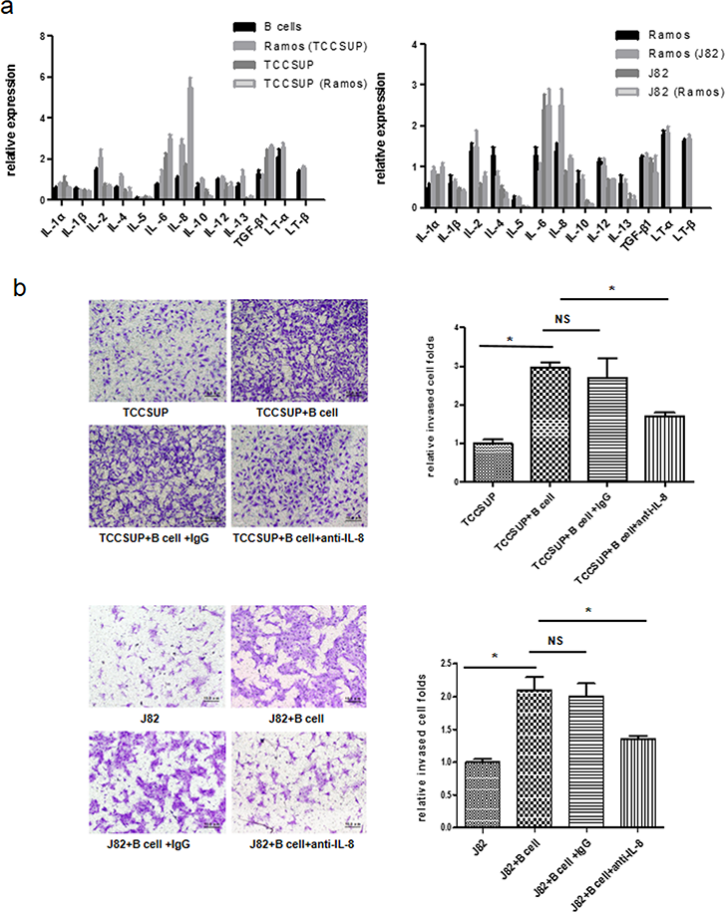

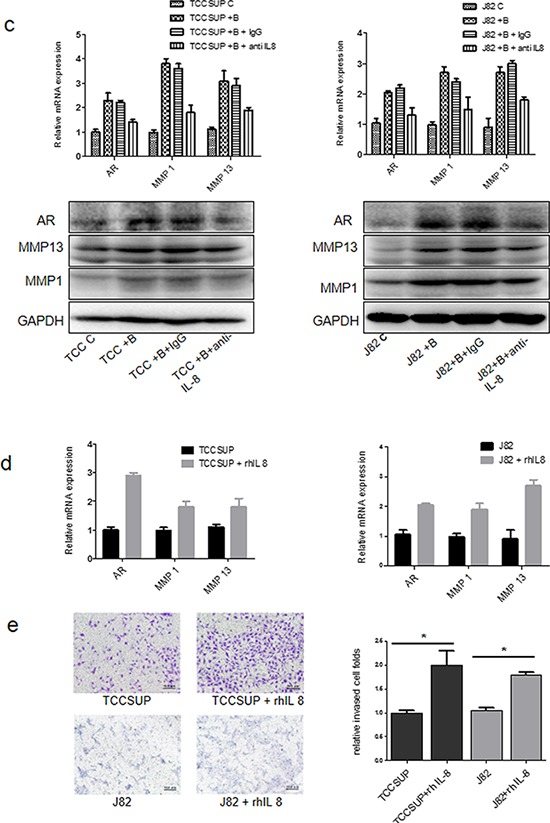
Mechanisms why infiltrating B cells could increase BCa cells AR expression with increased invasion **a.** qRT-PCR results showed IL-8 expressions were significantly increased in both B cells and BCa cells after co-culture Ramos (TCCSup) or Ramos (J82) stands for Ramos cells after co-culture with TCCSUP cells or J82 cells; TCCSUP (Ramos) or J82 (Ramos) stands for TCCSUP or J82 cells after co-culture with Ramos cells. **b.** B cells promotion of BCa cells invasion can be partly reversed by anti-IL-8 neutralizing antibody. **c.** qRT-PCR (upper panels) and Western blot (lower panels) results showed that blocking IL-8 using anti-IL-8 neutralizing antibody can partially reverse the B cells-mediated AR, MMP1 and MMP13 up-regulation in BCa cells. **d.** qRT-PCR results showed rhIL-8 can up-regulate AR, MMP1 and MMP13 expression in BCa cells. **e.** rhIL-8 can promote BCa cells invasion. (*P < 0.05).

The infiltrating immune cells may affect tumor progression through chemokines and cytokines production. So we focused on the secreted cytokines from infiltrating B cells to dissect the mechanism how infiltrating B cells may increase AR expression in BCa cells [13]. We screened several cytokines in B cells and BCa cells before and after co-culture, and found the IL-8 expression was consistently higher both in B cells and BCa cells after co-culture (Fig. 5a).

We then applied an interruption approach using 10 ug/ml anti-IL-8 neutralizing antibody to suppress secreted IL-8, and results suggested that blocking IL-8 partially reversed the B cells-increased BCa cell invasion in TCCSUP and J82 cells (Fig. 5b, *P < 0.05). Importantly, blocking IL-8 also reversed the increased expression of AR, MMP1 and MMP13 in BCa cells in the co-culture system (Fig. 5c).

Additionally, we applied another opposite approach *via* adding 20 ng/ml recombinant human IL-8 (rh-IL-8) in the co-culture system, and results revealed increased expression of AR, MMP1 and MMP13 expression in the BCa cells (Fig. 5d). Importantly, adding rh-IL-8 protein also increased BCa cells invasion (Fig. 5e).

Together, results from Fig. 5a–5e suggest that the IL-8/AR/MMP1/MMP13 signals might play key roles to mediate the infiltrating B cells-increased BCa cell invasion.

### Infiltrating B cells increased BCa metastasis in the *in vivo* mouse models

**Figure 6:**
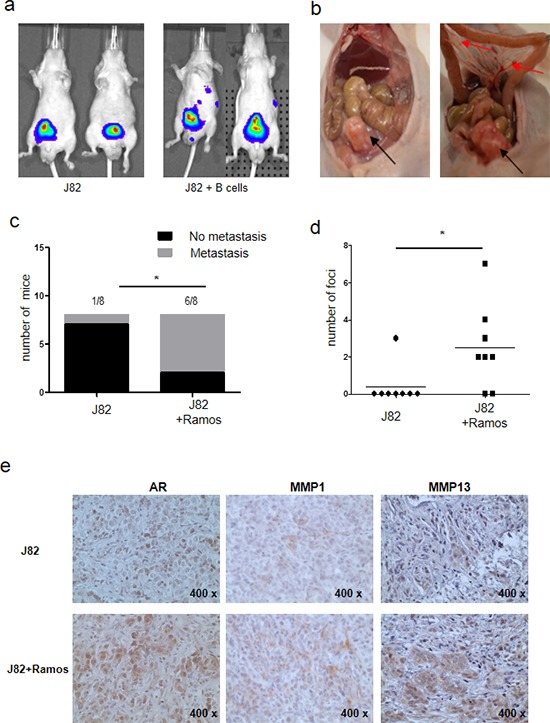
B cells promote BCa metastasis using *In vivo* orthotopic BCa model **a.** J82 cells were mixed with Ramos B cells and orthotopically implanted into bladders of nude mice. 6 weeks after implantation, the bladder tumors growth and metastasis were monitored by IVIS images. **b.** The images show bladder tumors (black arrows) and metastatic lymph nodes foci (red arrows). **c.** Quantification data for tumor metastases in mouse BCa model (*P < 0.05). **d.** Quantification data for tumor metastatic foci (*P < 0.05). **e.** IHC staining for AR, MMP1 and MMP13 in orthotopic bladder tumor tissues (400X).

To confirm the above *in vitro* results in the *in vivo* mouse model, we orthotopically xenografted nude mice with J82 cells co-implanted with B cells. The J82 cells were transfected with pCDNA3-luciferase for monitoring tumor growth and metastasis using the *in vivo* real-time imaging system (IVIS). 1 × 10^6^ J82 cells with or without 1 × 10^5^ B cells were implanted into the bladder wall. After 6 weeks, IVIS results revealed that 6 of 8 mice in the J82/B cells co-implanted group had metastatic luminescence signals located at distant locations. In contrast, metastatic foci were found in only one mouse of the J82 cells alone implanted group (Fig. 6a, 6c). The mice were then sacrificed for tumor confirmation, and results revealed that the metastatic foci were exclusively found in lymph nodes (Fig. 6b). None of the mice developed lung or liver metastasis. Importantly, we found more metastatic foci in J82 with B cells group than J82 cells alone group (20 vs 3, Fig. 6d). Results from IHC staining also indicated that the expressions of related key factors, including AR, MMP1 and MMP13, were much higher in J82/B cells co-implanted group than those found in J82 alone implanted group (Fig. 6e). These *in vivo* results matched well the data from *in vitro* co-culture studies.

## DISCUSSION

Previous epidemiological and preclinical studies demonstrated that androgens/AR signals play important roles in BCa development and progression. [3, 12] Early animal model studies also revealed that androgen/AR signals contributed to the bladder carcinogenesis, [14] and targeting AR signals may become a promising therapeutic option to treat BCa patients. [15]

B cells in the TME were found in various tumor samples, yet their pathological roles in the tumor progression remains controversial. Early studies from De Visser and his colleagues, using a transgenic mouse model, suggested that B cells could promote chronic inflammation to support carcinogenesis and tumor progression. [8] Other studies also indicated that B cells could increase cancer metastasis and promote castration-resistant prostate cancer through cytokine production. [16–18] In ovarian carcinomas, B cells might be able to predict a worse clinical outcome. [19] However, opposite effects were also reported showing B cells could suppress other types of tumor development, with higher tumor infiltrating B cell numbers correlated with a favorable prognosis. [20] B cells might exert their antitumor effects through collaborative interactions with T lymphocytes or by themselves. [21] In the present study, we used *in vitro* migration assays, invasion assays and *in vivo* BCa orthotopic mouse model to show that tumor infiltrating B cells could promote BCa metastasis.

IL-8 is a multifunctional cytokine that was originally identified as a neutrophil chemoattractant protein. IL-8 can be secreted by different cell types including lymphocytes, neutrophils, macrophages and many tumor cells. [22] Accumulating evidences showed that IL-8 level was increased in a variety of human tumors, such as colorectal cancer, breast cancer, prostate cancer and BCa. [23] Infiltrating monocytes and lymphocytes can further increase the level of IL-8 in the TME, [24] which may play key roles in tumor progression due to its ability to enhance the proliferation, invasion and angiogenesis of tumors. In BCa, IL-8 may be considered as a urinary biomarker for the detection of BCa and regulates tumorigenicity and metastasis in human BCa. [25, 26]

Interestingly, IL-8 has also been implicated in elevating the transcriptional activity of the AR and may promote prostate cancer progression *via* an androgen independent pathway. [27] Here we identified that IL-8 could be the AR up-stream signaling to modulate AR in BCa and recruitment of B cells to BCa cells could further increase the IL-8 expression.

MMPs are zinc-dependent endopeptidases involved in extracellular matrix (ECM) degradation and play an important role in the process of carcinogenesis, cell adhesion, epithelial–mesenchymal transition and tumor angiogenesis. [28] MMP1 and MMP13 belong to the collagenase subgroup of MMPs, which are capable of degrading fibrillar collagens in the extracellular space. It is well known that MMP1 and MMP13 are strongly related to tumor invasion and metastatic abilities. [29] Some reports also show that MMP1 and MMP13 play important roles in BCa progression. [30, 31] Here we proved that the infiltration of B cells towards BCa cells could increase MMP1 and MMP13 expression that might then result in increased BCa invasion.

In summary, our results demonstrated that infiltrating B cells could enhance BCa invasion and metastasis *via* modulation of IL-8/AR/MMPs signals. Future studies may allow us to develop an effective therapeutic strategy to interrupt these newly identified B cells/IL-8/AR/MMPs signals to better suppress BCa metastasis.

## MATERIALS AND METHODS

### Patients

Bladder tumor specimens and adjacent normal bladder tissues were collected from 24 patients who were diagnosed with BCa in Xiangya Hospital, Central South University, Changsha, China. All these patients were treated with surgery and no other therapy before surgery.

### Cell lines

Normal human urothelial cell line SVHUC, human BCa cell lines TCCSUP, T24 and J82 and the Ramos B cell line were purchased from the American Type Culture Collection (ATCC, Manassas, VA). SVHUC cells were cultured in Kaighn's Modification of Ham's F-12 Medium supplemented with 10% fetal bovine serum (FBS). TCCSUP, T24 and J82 cells were cultured in Dulbecco's Modified Eagle Media (DMEM) supplemented with 10% FBS. B cells, which grow in suspension culture, were cultured in Roswell Park Memorial Institute (RPMI) 1640 medium supplemented with 10% heat-inactivated FBS, 2 mM L-glutamine, 100 IU/mL penicillin, 50 μg/mL streptomycin. Cells were maintained in a humidified 5% CO2 environment at 37°C.

### Reagents and materials

Anti-GAPDH (6c5), and anti-AR antibodies were purchased from Santa Cruz Biotechnology (Santa Cruz, CA). Anti-MMP1 and Anti-MMP13 antibodies were purchased from Bioss (bs-4597R, bs-0575R, Woburn, MA). Anti-Human IL-8 antibody was purchased from Peprotech (500-p28, Rocky Hill, NJ). Recombinant Human IL-8 was purchased from R and D Systems (208-IL-010, Minneapolis, MN). MMP1 inhibitor was purchased from EMD Millipore (#444250, Gibbstown, NJ) and MMP13 inhibitor was purchased from Sigma (CL-82198, St Louis, MO). Polyvinylidene defluoride membrane (PVDF) was from Thermo Fisher Scientific (Rochester, NY). Anti-mouse/rabbit secondary antibody for Western blot was from Invitrogen (Grand Island, NY).

### B cells recruitment assay

The recruitment assay was performed in a 24-well transwell system with 5 μm pore polycarbonate membrane inserts (Corning, #3422, Corning, NY, USA). Conditioned media (CM) of BCa cells or SVHUC cells were plated into the lower chambers of the transwells. 1 × 10^5^ B cells were plated into the upper chambers. After 6 hrs, the B cells migrated into the lower chamber media were collected and counted.

### Migration and invasion assays

Cell migration assays and invasion assays were performed using the transwell system (Corning, 8 mm pores). The upper chamber inserts remained uncoated for migration assays or coated with diluted matrigel (BD Biosciences, Sparks, MD) for invasion assays. Before performing migration and invasion assays, BCa cells were cultured with B cells for 72 hrs. 1 × 10^5^ BCa cells (in serum free media) and 10% serum containing media were plated in the upper and lower chambers, respectively. After 24 hrs incubation migrated/invaded cells invaded cells were stained with 0.1% crystal violet, and positively stained cells were counted. The cell numbers were obtained by counting six random fields. Quantitation indicates Mean ± SD of triplicate repeats.

### 3D invasion assay

Matrigel was thawed on ice and added to each well of 8-well glass chamber slides (at 50 μl/cm^2^), was spread evenly and the slides placed in the cell culture incubator to allow the Matrigel to solidify (takes 15 to 20 min). 1 × 10^5^ J82 cells were placed into each well after co-culture with B cells with media containing 5% Matrigel and10 ng/ml epithelial growth factor (EGF). T24 and J82 cells take about 10 days to form acini-like structures. 10 different random fields under microscope were chosen and the number of structures in each field counted.

### RNA extraction and quantitative real-time PCR analysis

Total RNAs were isolated using Trizol reagent (Invitrogen, Grand Island, NY). 2 μg of total RNA was used for reverse transcription using Superscript III transcriptase (Invitrogen). Quantitative real-time PCR (qRT-PCR) was conducted using a Bio-Rad CFX96 system with SYBR green to measure the mRNA expression level of a gene of interest. Expression levels were normalized to the expression of GAPDH RNA.

### Western blot analysis

Western blotting was performed as previously described. [11] Briefly, cells were washed with PBS and then lysed in RIPA buffer. Quantified proteins were separated on 10% sodium dodecyl sulfate (SDS)-polyacrylamide gel electrophoresis (PAGE) and then transferred onto PVDF membranes. After blocking membranes, they were incubated with appropriate dilutions of specific primary antibodies. After washing in Tris-buffered saline plus 0.05% Tween-20 (TBS-T), the blots were incubated with peroxidase-conjugated secondary antibodies and visualized using enhanced chemiluminescence system (Thermo Fisher Scientific).

### Lentivirus packaging and transfection

We designed the AR shRNA sequences that which are compatible with the PLKO1.0 vector. psPAX2 and pMD2.G were applied as packaging and envelop plasmids, respectively. The plasmids were transfected into 293T cells. After 48 hrs, the lentivirus soup was harvested for immediate use or frozen for later use. The collected viruses were added to the target cells to incubate for 24 hrs. Cell cultures were refreshed with culture media and cultured for another 3 days to allow target protein expression.

### Luciferase reporter assays

Promoters of MMP1 and MMP13 were obtained from genomic DNA of HEK293T cells by Phusion^®^ High-Fidelity DNA Polymerase (NEB, Rochester, NY) and constructed into pGL3-basic vector (Promega, Madison, WI) by Gibson assembly method. Cells were plated in 24-well plates and transfected with cDNA using Lipofectamine 3000 (Invitrogen). Thymidine kinase promoter-Renilla luciferase reporter plasmid (pRL-TK) was used as the internal control. Luciferase activity was measured by Dual-Luciferase Assay (Promega) according to the manufacturer's manual.

### Chromatin immunoprecipitation assay (ChIP)

Cells were cross-linked with 1% formaldehyde and glycine was added to stop the reaction 10 min later. The cells were then washed with PBS and resuspended in lysis buffer. Sonication was used to shear chromatin to obtain 200–500 bp DNA fragments. Anti-AR antibody (Santa Cruz) was used for immunoprecipitation. IgG was used as negative control. Specific primers were designed to amplify target sequences with human MMP1 and MMP13 promoters. PCR products were identified by agarose gel electrophoresis.

### *In vivo* metastasis studies

Male 6–8 weeks old nude mice were purchased from NCI. J82 cells were transfected with luciferase reporter gene (pcDNA3.0-luciferase) and the positive stable clones were selected and expanded in culture. Orthotopic xenografts of these BCa cells were applied according to a previous report. [32] Briefly, after mice were anesthetized, a lower mid-line abdominal incision was made, and the bladder was exposed. BCa cells resuspended in PBS and mixed with matrigel (1:1) were injected into the bladder wall muscle. In the control group, 8 mice were implanted with 1 × 10^6^ BCa cells only, while in co-culture group, 8 mice were co-implanted with 1 × 10^6^ BCa cells and 1 × 10^5^ B cells. Fluorescent Imager (IVIS Spectrum, Caliper Life Sciences, Hopkinton, MA) was applied to monitor metastasis in live mice every week after tumor cell implantation and following injection of 150 mg/kg Luciferin. After 6 weeks, mice were sacrificed and the primary tumors and metastatic sites were further examined. All animal studies were performed under the supervision and guidelines of the University of Rochester Medical Center Animal Care and Use Committee and followed approved protocols.

### Immunohistochemical staining

Immunohistochemical staining was performed on the samples from the human BCa tissues and mouse xenografted tumors. The samples were fixed in 4% neutral buffered para-formaldehyde, embedded in paraffin, and cut into 5 μm slices. After deparaffinization, hydration, and antigen retrieval, these sections were incubated with corresponding primary antibodies, incubated with biotinylated secondary antibodies (Vector, Burlingame, CA) and then visualized by VECTASTAIN ABC peroxidase system and 3, 3′-diaminobenzidine (DAB) kit (Vector).

### Statistical analysis

All data are presented as mean ± SD from at least 3 independent experiments. Statistical analyses were performed with SPSS 17.0 (SPSS Inc., Chicago, IL). Differences between 2 groups were analyzed by paired Student's *t* test. *P* < 0.05 was considered statistically significant.

## SUPPLEMENTARY FIGURES


