# Supplementary material for: Tumor microenvironment B cells increase bladder cancer metastasis via modulation of the IL-8/androgen receptor (AR)/MMPs signals
Source: Oncotarget. 2015 Jul 17;6(28):26065–78. doi: 10.18632/oncotarget.4569 (PMC4694886; doi:10.18632/oncotarget.4569)
Supplement: Supplementary file 1 [file oncotarget-06-26065-s001.pdf]

SUPPLEMENTARY FIGURES

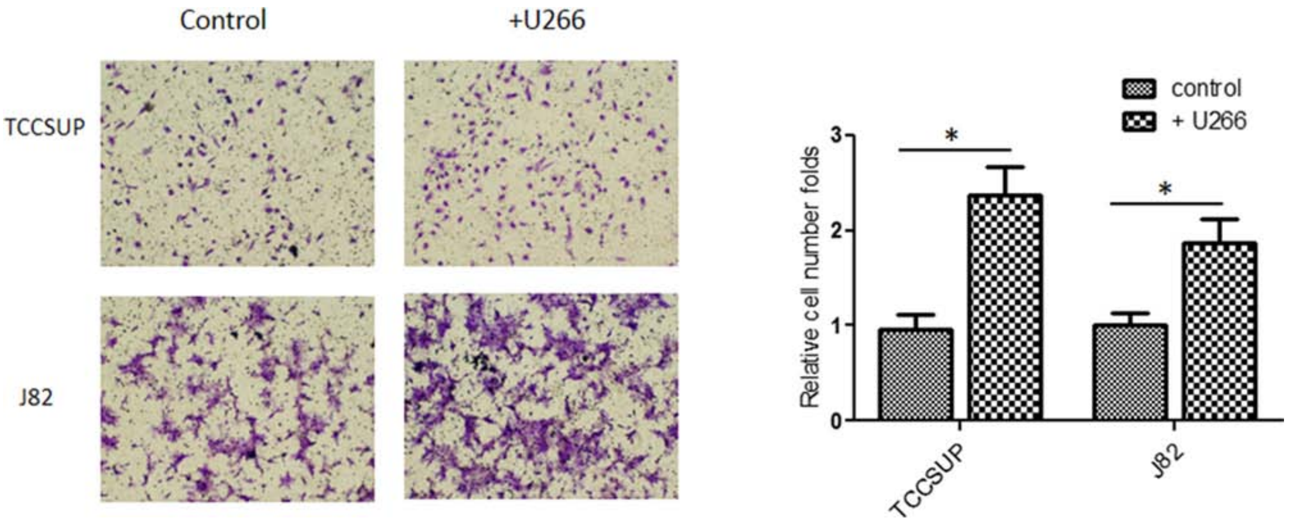

Supplementary Figure S1: Invasion assay of BCa cells after co-culture with U266 cells. TCCSUP and J82 cells were co-cultured with U266 cells for 3 days. Image shows BCa cells co-cultured with U266 cells have a higher invasiveness. The right panel is the quantification data of changed BCa invasion abilites. *$p < 0.005$.

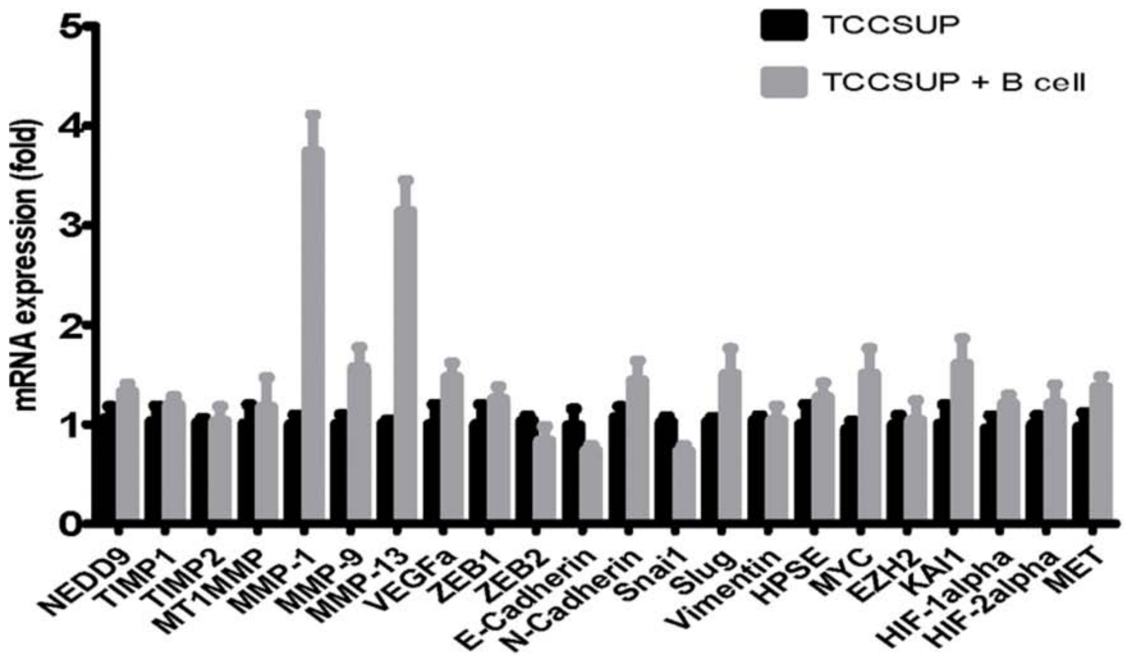

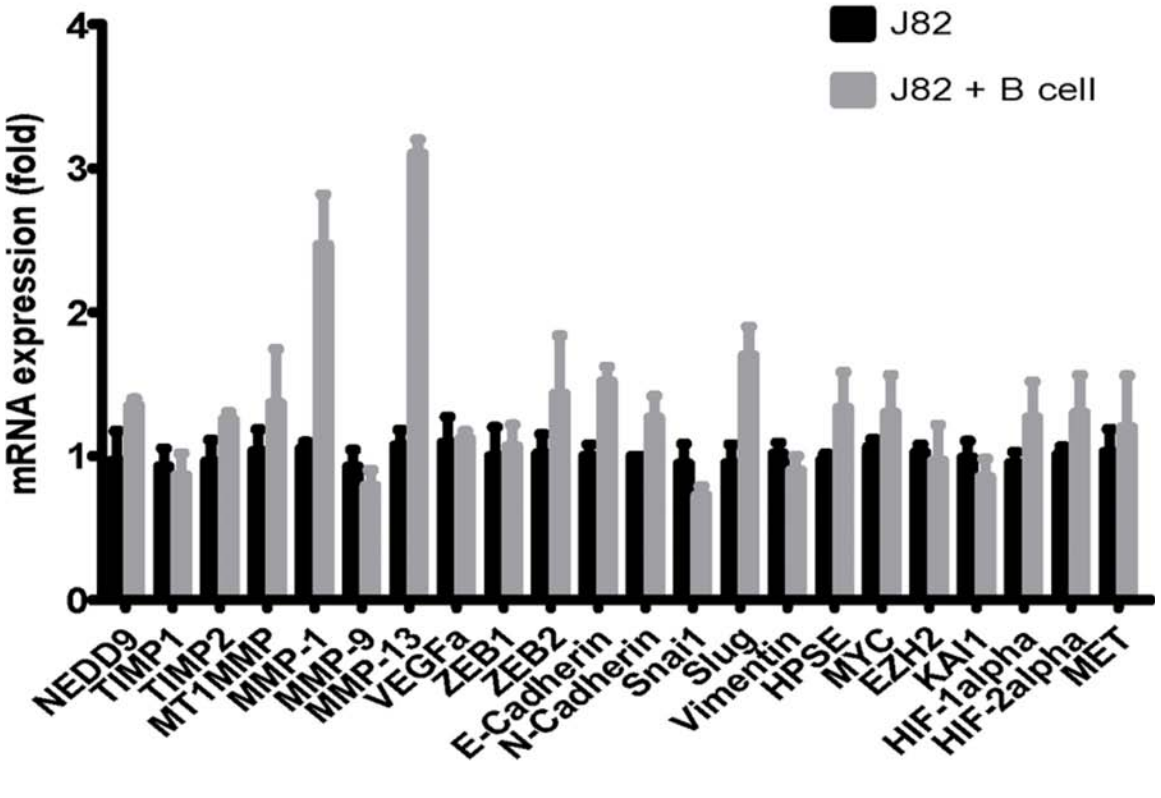

Supplementary Figure S2: Quantitative real-time PCR shows mRNA level changes of BCa cells after co-culture with Ramos cells.

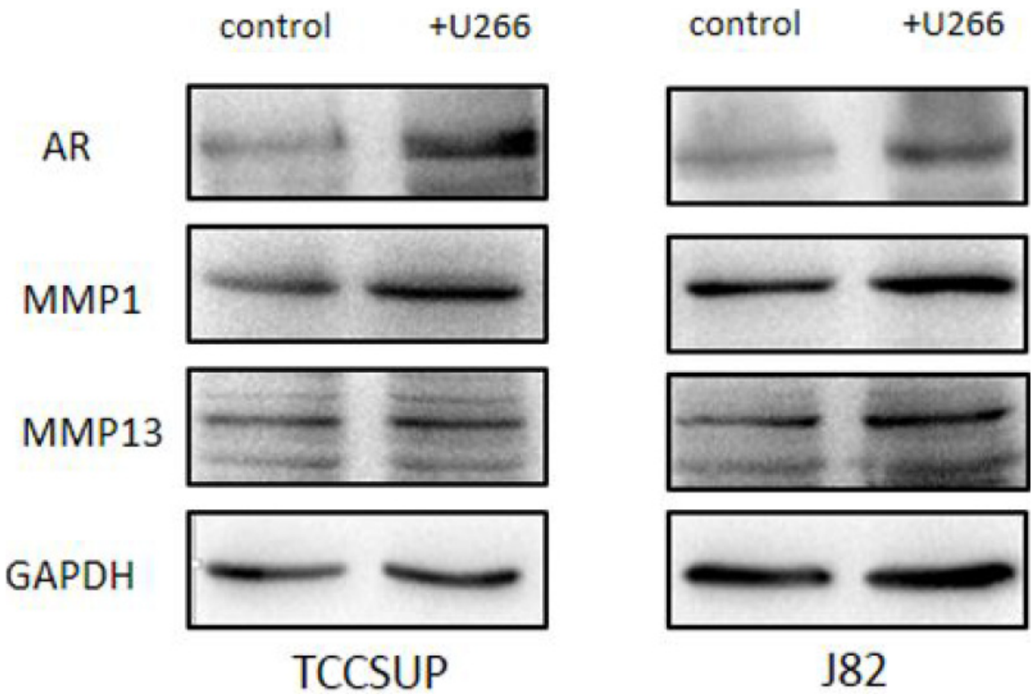

Supplementary Figure S3: Protein level change of BCa cells after co-culture with U266 cells. Westen blot results show AR, MMP1 and MMP13 protein expression in TCCSUP and J82 cells were increased after co-culturing with U266 cells.
